# How to Get the Best from Low-Cost Particulate Matter Sensors: Guidelines and Practical Recommendations

**DOI:** 10.3390/s20113073

**Published:** 2020-05-29

**Authors:** Erika Brattich, Alessandro Bracci, Alessandro Zappi, Pietro Morozzi, Silvana Di Sabatino, Federico Porcù, Francesca Di Nicola, Laura Tositti

**Affiliations:** 1 Department of Physics and Astronomy, Alma Mater Studiorum University of Bologna, 40126 Bologna, Italy; alessandro.bracci5@unibo.it (A.B.); silvana.disabatino@unibo.it (S.D.S.); federico.porcu@unibo.it (F.P.); francesca.dinicola2@unibo.it (F.D.N.); 2 Department of Chemistry “G. Ciamician”, Alma Mater Studiorum University of Bologna, 40126 Bologna, Italy; alessandro.zappi4@unibo.it (A.Z.); pietro.morozzi2@unibo.it (P.M.); laura.tositti@unibo.it (L.T.)

**Keywords:** low-cost sensors, air quality, particulate matter, optical particle counter, particle mass concentration, PM10, PM2.5, PM1, particle size distribution

## Abstract

Low-cost sensors based on the optical particle counter (OPC) are increasingly being used to collect particulate matter (PM) data at high space and time resolution. In spite of their huge explorative potential, practical guidelines and recommendations for their use are still limited. In this work, we outline a few best practices for the optimal use of PM low-cost sensors based on the results of an intensive field campaign performed in Bologna (44°30′ N, 11°21′ E; Italy) under different weather conditions. Briefly, the performances of a series of sensors were evaluated against a calibrated mainstream OPC with a heated inlet, using a robust approach based on a suite of statistical indexes capable of evaluating both correlations and biases in respect to the reference sensor. Our results show that the sensor performance is sensibly affected by both time resolution and weather with biases maximized at high time resolution and high relative humidity. Optimization of PM data obtained is therefore achievable by lowering time resolution and applying suitable correction factors for hygroscopic growth based on the inherent particle size distribution.

## 1. Introduction

The assessment of high-resolution air quality data in highly inhomogeneous areas such as the urban environment is a major requirement for the understanding of the complex physico-chemical processes behind the accumulation of atmospheric pollutants. This is especially true for airborne particulate matter (PM), a pollutant characterized by multiple emission sources simultaneously active at a single place; complex chemical phenomenology affects both composition and particle size, strongly affected not only by local advection pattern but also by local turbulence.

In order to comply with governmental air quality standards, monitoring stations are deployed and managed by regional environmental protection agencies which collect systematic and routine data of particle and gaseous pollutants using highly reliable but costly, large and usually static certified reference instruments, according to the environmental prescription and regulations [1]. Due to the high costs of the installation and maintenance of reference monitoring stations in terms of economic and human resources, this approach is limited to a few sparse stations over single urban areas. The urban canopy is highly complex in terms of spatial patterns [2], topography, dispersion and deposition conditions [3], and emission profiles, which induce localized gradients in ground-level concentrations of air pollutants [4]. Therefore, the available monitoring stations, though satisfying the legislative requirements, are hardly representative of the whole urban surface, preventing an accurate assessment of spatially resolved environmental conditions and the inherent risks for the population. Environmental protection agencies usually complement air quality monitoring from static monitoring stations with mobile monitoring stations sampling additional local sites not covered by the reference network for fixed periods [1]. Mobile laboratories use the same instrumentation as the permanent monitoring stations, and therefore suffer the same high maintenance and calibration costs. In addition, though data collected from mobile laboratories are precious in terms of accuracy and for specific purposes under intensive field campaigns, they are affected by significant temporal limitations, not representative of the whole annual and interannual variability. For this reason, the deployment of low-cost sensors networks is becoming increasingly popular, allowing not only integration of the environmental information at a high space and time resolution but also raising citizen awareness [1,5] and involvement in the management of environmental issues [6]. In addition, recent approaches to complement data from low-cost sensors in air quality models have been proposed to increase spatial and temporal resolution and reduce biases and systematic errors [7,8,9]. The success of these initiatives within the broader framework of IoT (Internet of Things) is very promising; however, from a metrological standpoint, one of the main drawbacks of this approach is the lack of a suitable evaluation of the consistency and reliability of the experimental data collected with low cost sensors, a non-negligible aspect for the subsequent data elaborations and deductions.

In the case of PM sensors, many factors should be accounted for, owing to a series of intrinsic and extrinsic factors. On the global scale, PM metrics included in the air quality networks rely on the official method (EN 12341:2014), which consists of the collection of PM10 and PM2.5 usually on quartz fiber filters by sequential certified samplers and successive gravimetric analysis, or by continuous detection based on beta-attenuation technique or tapered element oscillating microbalance (TEOM) [10,11]. However, scientists widely agree that a more informative and accurate approach should also include the assessment of the corresponding size distribution closely related to PM atmospheric residence time, transport and health effects [12,13,14]. In this framework, the use of optical particle counters (OPCs) is highly valued since they allow for the collection of information on particle size distributions and for the conversion of number densities obtained with the aid of suitable parameterizations and assumptions usually based on empirical data into mass concentration data easily interpretable as a function of air quality standards.

While OPCs are widely used instruments in atmospheric research, they are not routinely employed in air quality networks. Therefore standardization protocols are not widely applied, even if recently the ISO 21501 (and in particular the recent Part 4:2018 Determination of particle size distribution—single particle light interaction methods—light scattering airborne particle counter for clean spaces) has been made available after the dismission of ASTM F 328-98(2003). This standard method is usually carried out by the manufacturer. As a result, owing to the need for special experimental facilities, even research-oriented instruments usually rely solely on calibration procedures carried out at the manufacturers with a frequency dictated by a single laboratory’s responsibility and awareness.

Therefore, even research-based instrumentation poses some fundamental questions in terms of PM data quality and assimilation to be employed in whatever environmental application.

Low-cost PM sensors are based on the same technology. In order to obtain reliable data, it is necessary to evaluate their uncertainties, performance and accuracy with laboratory tests and field comparisons with reference instruments [15].

Further sources of uncertainty in PM data assessments arise from the meteorological variability, since different weather and dispersion conditions may change the physico-chemical nature of the particles observed at the receptor site over time. In particular, the variability of wind circulation and boundary layer mixing affect the ventilation, dilution and upwind emission sources [16]. Temperature and relative humidity conditions affect the stability of aerosol components, such as NH_4_NO_3_ and several carbonaceous components, and the kinds and rates of chemical reactions, leading to the formation of secondary inorganic and organic aerosols [17,18,19]. In addition, atmospheric fluctuations in relative humidity (RH) which in turn depend not only on the air masses transiting over a given place but also on the setting of different thermodynamic and microphysical conditions, are capable of modifying the particle size distribution sensibly as a result of aerosol hygroscopicity [20].

Recently, several papers focusing on the evaluation of the affordability of low-cost sensors from laboratory tests and experimental field campaigns have been published [21,22,23,24,25,26,27,28,29,30]. However, previous studies often failed in deriving practical guidelines and recommendations for the best practices with which to use low-cost sensors in terms of time resolution and different weather conditions. In this work, we present the results of an intercomparison exercise performed during a long-term (more than six months of monitoring) experimental field campaign aimed at assessing the performance of a series of low-cost sensors in terms of accuracy and reproducibility under different time resolution and weather conditions.

As such, this work aims to:

- Characterize the performances and reproducibility of different brands of low sensors in comparison to reference instruments;
- Assess instrument variability using batches of the same kind of low-cost sensors from the same producer;
- Perform a comparative analysis of the various OPCs under different meteorological conditions capable of sensibly affecting the PM size distribution, and consequently, the estimated mass concentration data.

This work is organized as follows. Following the Introduction section, Section 2 presents the material and methods used in this work, including the site where the experimental field campaign was performed, the instrumentation adopted, the algorithm used to convert number densities to mass concentration and the statistical parameters used to evaluate the performances of low-cost sensors. Section 3 presents and discusses the results in terms of particulate matter concentrations and particle number densities, and finally, Section 4 draws the main conclusions of the work.

## 2. Materials and Methods

In this work, we evaluated the performances of a set of different low-cost and research-oriented laser particle counters in outdoor ambient conditions during two distinct experimental field campaigns performed during the periods June 6th 2019–August 4th 2019 and September 23rd 2019–February 12th 2020 in Bologna (44°30′ N, 11°21′ E; Northern Italy), a city located in the Po Valley, a region representing one of the major pollution hotspots in Europe (e.g., [31,32]). The study periods included a range of different meteorological conditions, well representative of the typical weather conditions affecting the city and the surrounding region in the warm and cold seasons.

Bologna is characterized by a humid temperate climate (Cfa following Köppen climate classification), with quite cold, humid and damp winters and hot and muggy summers. Humidity is generally quite high during both summer and winter periods, while the wind circulation is characterized by frequent stagnation conditions under calm and breeze regimes. Precipitation is moderate and quite well distributed over the seasons, with two peaks in spring and autumn and relative minima in winter and summer.

### 2.1. Instrumentation

For the purpose of investigating and comparing the performances of the sensors in measuring particle size distributions and particle mass concentrations, they were co-located on the rooftop of the Department of Physics and Astronomy of the University of Bologna. Optical sensors included: one Profiler Model 212 (MetOne Instruments, Inc., Grants Pass, OR 97526, USA), a couple of OPC-N2 low-cost sensors (Alphasense Ltd., Braintree CM77 7AA, UK; price around 300–400 €), a couple of iSCAPE Citizen Kits (SCK, Fab Lab Barcelona 08002, Barcelona, Spain; based on the Smart Citizen Kit 2.0 and PMS 7003 particle sensors, price around 100–200 €) low-cost sensors and one LOAC (light optical aerosol counter; MeteoModem, 77760 Ury, France).

All the sensors utilized in this work are optical particle monitors that use laser beams to detect and count particles, thereby evaluating the scattering signal from suspended particulate to provide a semi-continuous real-time measurement of airborne particulate as a function of size. In particular, the MetOne Profiler 212 is a robust mainstream optical particle counter widely employed in air monitoring (see for example [33,34]), regularly re-calibrated on an annual basis and employed in this work as a reference instrument; i.e., following a heating step causing particle dehydration and classification of particles based on their dry diameters.

The OPC-N2 and the SCK sensors are low-cost sensors characterized by a low price, extreme portability and small weight (e.g., [22,29,30]). According to [35], the low-cost sensors herein evaluated can be classified respectively as OEM (original equipment manufacturers) and SSys (sensor systems); i.e., equipment based on low-cost sensors mounted by the customers or already available in a “ready-to-use” format. The Alphasense OPC-N2 sensors were mounted at our lab in a waterproof case together with other meteorological and gaseous pollutant sensors not treated in this work; the whole system (ABBA; Arduino Board Based Air quality monitoring system) was designed and built within the team authoring this paper.

Finally, the LOAC is a versatile optical particle counter characterized by low weight and extreme compactness, which enables it to perform measurements not only at the surface but dynamic applications such as balloon platforms in the troposphere and the stratosphere [36,37].

The optical counters used in this work are characterized by different size ranges, flow rates, time resolution, scattering angles and laser wavelengths; all the devices use a mirror to ensure the photodetector collects efficiently all scattered light. In the case of LOAC, the size distribution is estimated from two scattering angles: one around 12° almost insensitive to the refractive index of the particles and one around 60° strongly sensitive to the refractive index of the particles [36]. This enables the sensor to determine not only the size distribution but also the typology of the dominant aerosol (droplets, carbonaceous, salts and mineral particles). An important characteristic of an optical particle counter is the counting efficiency; i.e., the maximum number of particles or maximum concentration of particles that can be detected by the sensor [38]. The counting efficiency of the sensors used in this work is quite variable and also depends on the diameter and meteorological conditions for the LOAC. Table 1 summarizes the main technical specifications of the sensors used in this work.

The measurement time resolution of the MetOne Profiler 212 (hereafter MetOne), OPC-N2 and LOAC sensors was set to 60 s. Instead, the SCKs made measurements integrated over 30 s, which were averaged to the same time resolution of the other sensors for the sake of homogeneity.

Observations of meteorological variables (atmospheric pressure, air temperature, air relative humidity, wind speed and wind direction) were collected by a Davis Vantage Pro2 (Davis Instruments, Hayward, CA 94545, USA) on a 10-min time basis.

### 2.2. Number Concentration to Mass Conversion

Optical particle counters are typically based on light scattering by aerosol particles in a flow cell (e.g., [39,40,41]). As a result, this broad family of instruments is designed to assess particle number densities as a function of particle size bins based on suitable scattering parameterization. Particle number density is, therefore, a fundamental metric to integrate efficiently aerosol gravimetry-based metrics. The MetOne herein used as a reference provides, therefore, the number densities in the particle size intervals previously detailed. In order to make all the data comparable, signals from the MetOne were converted into mass concentration (μg m^−3^).

Conversely, as previously described, the OPC-N2 and the SCK sensors estimate PM1, PM2.5 and PM10 mass concentrations from count measurements using embedded proprietary algorithms not yet disclosed to the public. Similarly, the LOAC sensor estimates PM2.5 and PM10 mass concentrations from count measurements. In general, the algorithms used by the optical sensors assume a default particle density (1650 kg m^−3^ in the case of OPC-N2; unknown for the SCKs); a volume-weighting factor (default set to 1) to account for errors in sizing due to differences in the refractive index of particles used for calibration and those being measured [42]; and for SCK, an atmospheric correction factor used in field evaluation whose details are not available from the manufacturer.

Number density data obtained by the MetOne was therefore subjected to a suitable inversion procedure for the planned comparison with the other devices. The following computation was used based on the assumptions of spherical particles with uniform density, a standard approximation, though actually not complying with real ambient aerosol particles [43].

For each size bin of the instrument, a weighted volume diameter was computed according to [43]:

(1) $$D=\mathrm{LB}{\left[\frac{1}{4}\left(1+{\left(\frac{\mathrm{UB}}{\mathrm{LB}}\right)}^{2}\right)\left(1+\frac{\mathrm{UB}}{\mathrm{LB}}\right)\right]}^{1/3}$$

where *LB* and *UB* are respectively the lower and upper boundaries of each size bin. The particle volume (*V_p_*) and mass are then calculated as follows:

(2) $$V_{p}=\frac{\pi D^{3}n}{6}$$

(3) $$m={\rho V}_{p}$$

where *n* is the particle count and *ρ* is the particle density in g cm^−3^. According to [44,45], the value of particle density was assumed equal to 1.65 g cm^−3^, a widely accepted approximation well representative of urban average particle mixture [43,45]. The particle mass is eventually divided by the sampled air volume to provide aerosol mass concentration per unit air volume. Note that in the case of the LOAC instrument, *m* is not the particle mass, but it is already the volume mass concentration.

Particle mass concentrations for the MetOne (PM1, PM2.5, and PM10) and the LOAC (PM1) were finally estimated by summing the concentration masses in the various size bins fitting to the respective aerosol cutoff.

### 2.3. Sensor Performance Metrics

For the purpose of investigating the performances of the OPC-N2, SCK and LOAC particle sensors, their measurements were compared to those obtained from the co-located MetOne instrument. In particular, the choice to use the measurements retrieved from the MetOne as the reference values derived from the fact that this instrument is widely used internationally and therefore fairly well characterized, at least compared to the others (e.g., [33,46,47,48,49,50]). This instrument is endowed with a humidity sensor and an inlet heater to prevent moisture from being sampled as particulate mass.

In order to detect and remove outliers from the 1-min time series recorded by all the sensors, we used the Hampel filter based on the calculation of the median and the standard deviation expressed as median absolute deviation (MAD) over a sliding window [51,52]. The filter identifies as outliers values differing from the window median by more than *x* standard deviations and substitutes them with the median. The filter has two configurable parameters; namely, the size of the sliding window and the number of standard deviations which identify the outlier (*x)*. In this case, we selected a sliding window of 7 observations (given observation and the 2 × 7 surrounding elements) and a value of 2 × MAD to detect outliers.

In particular, besides visual comparisons of the time series, scatterplots and histograms produced with the “psych” package [53] for the R language, version 3.6.1 [54] and density scatter plots built using “ksdensity” Matlab function, a set of statistical indexes was calculated. Indexes include widely used metrics (e.g., [55]), such as the mean bias error (*MBE*), the mean absolute error (*MAE*), the root mean square error (*RMSE*), the correlation coefficient (*r*), the coefficient of determination (*R^2^*) and the t-score (*t*). In addition, normalized values were also calculated for MBE, MAE and RMSE, using the observation range (*NMBE*, *NMAE* and *NRMSE*) or the observation average (*CVMBE*, *CVMAE* and *CVRMSE*) as a factor for normalizations. Calculations were performed using the “tdr” package [56] for the R language. Formulas are provided in Appendix A. Our approach, using a combination of qualitative and quantitative analyses including statistical parameters such as the bias, the RMSE and the MAE, besides the correlation coefficient, is very robust and complete for characterizing the performances of the sensors completely [35].

## 3. Results and Discussion

In this study, we evaluated the performances of a suite of particle sensors in terms of mass concentrations and particle number densities, as evaluated from the comparison with a co-located reference instrument under different sampling conditions. This section presents and discusses the main results of this intercomparison exercise, focusing firstly on mass concentrations and secondly on particle number densities to better understand the reasons for the different behaviors.

### 3.1. Particle Mass Concentrations

#### 3.1.1. Effect of Seasonal Variability

**Figure 1:**
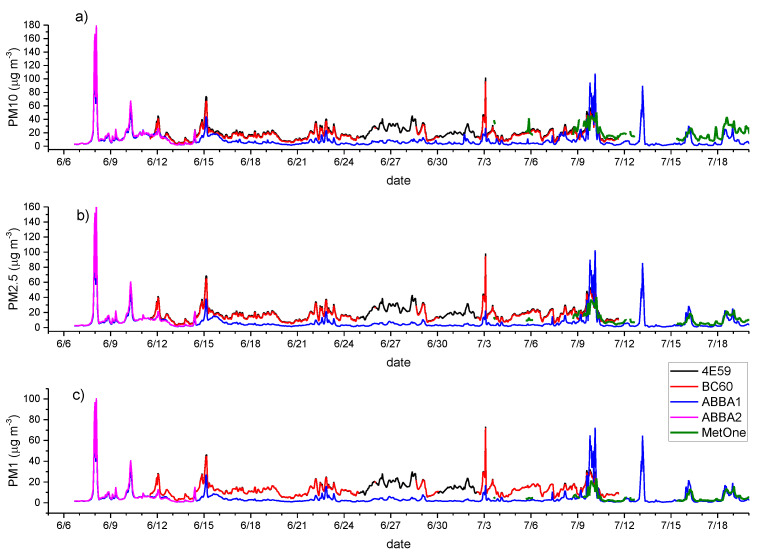
Comparison of hourly PM10 (**a**), PM2.5 (**b**) and PM1 (**c**) (µg m^−3^) mass concentrations from the co-located particle sensors during the summer measurement period. 4E59 (black line) and BC60 (red line) are the two SCK (Smart Citizen Kit) sensors; ABBA1 (blue line) and ABBA2 (pink line) are the two OPC-N2 sensors from Alphasense. MetOne (thick green line) is the reference instrument.

**Figure 2:**
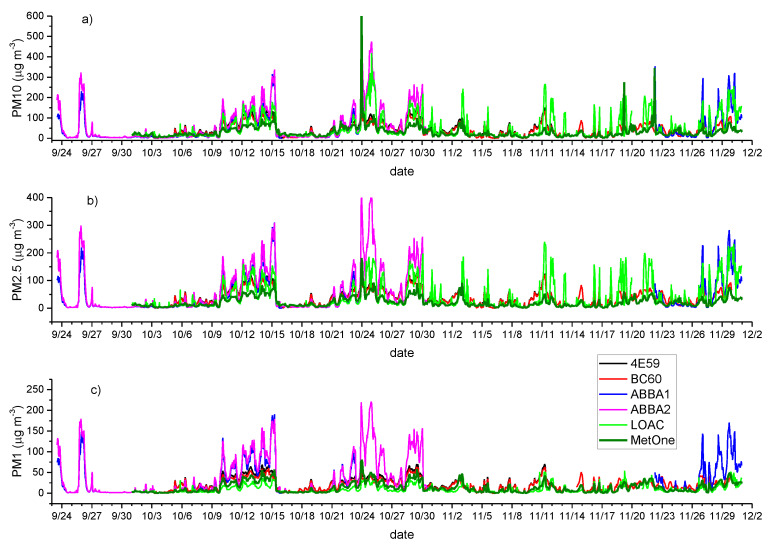
Comparison of hourly PM10 (**a**), PM2.5 (**b**) and PM1 (**c**) (µg m^−3^) mass concentrations from the co-located particle sensors during the autumn measurement period. 4E59 (black line) and BC60 (red line) are the two SCK sensors; ABBA1 (blue line) and ABBA2 (pink line) are the two OPC-N2s sensors. LOAC (Light Optical Aerosols Counter) is the light green line. MetOne (thick olive green line) is the reference instrument.

Figure 1 and Figure 2 present the time series of hourly particle mass concentrations (PM10, PM2.5 and PM10) observed by the two SCKs (4E59 and BC60), the two OPC-N2s (ABBA1 and ABBA2), the LOAC (during autumn) and the MetOne during the summer, autumn and winter experimental campaigns. Notwithstanding the application of the Hampel filter, as detailed in Section 2, Figure 1 and Figure 2 highlight clearly that data collected from low-cost sensors in both seasons and from the LOAC during autumn are characterized by evident spikes in mass concentrations, usually not observed or at least less evident in the observations from the MetOne instrument. In addition, biases between the two types of low-cost sensors and the reference instrument are larger during the summer season (Figure 1) than during autumn (Figure 2). During both seasons, the most significant biases affect PM1, while biases seem to be reduced for PM10. Finally, Figure 1 and Figure 2 highlight that the two couples of SCKs and OPC-N2s are characterized by high correlations and very similar temporal patterns.

Comparisons of hourly observations of PM2.5/PM10 and PM1/PM2.5 concentration ratios are reported in Appendix B. The hourly resolution of these time series has been chosen with respect to the 10-min resolution to improve the graphic representation of the long-term time series. This comparison highlights that, in general, the two SCKs are characterized by very high concentration ratios with respect to the MetOne, while observations from the OPC-N2s show a better agreement with those from MetOne, particularly as far as the PM2.5/PM10 ratio is concerned. The very high ratios observed by the two SCKs might derive from the reduced particle-size selectivity of the Plantower PMS5003 particle sensor of the SCKs, as evidenced by [25].

**Figure 3:**
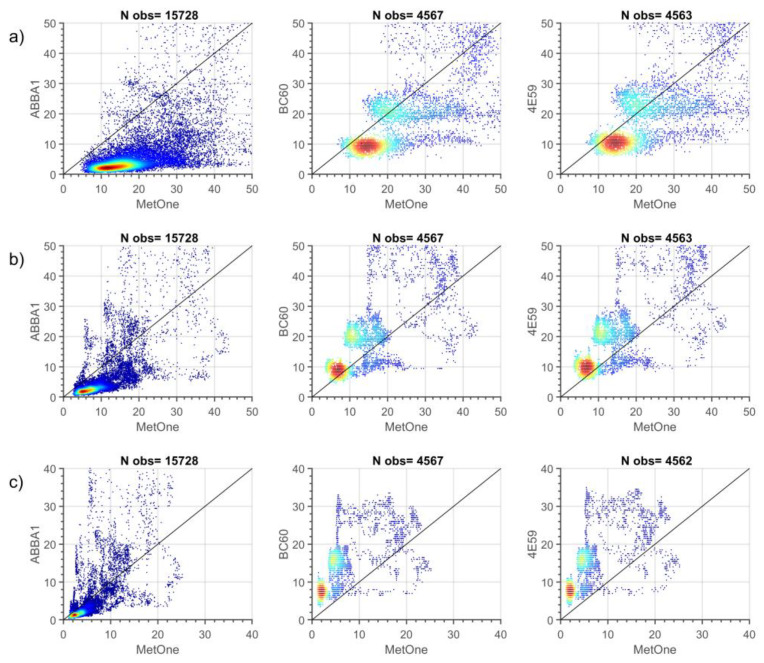
Density scatter plot for of 1-min PM10 (**a**), PM2.5 (**b**) and PM1 (**c**) (µg m^−3^) mass concentrations from the co-located particle sensors during the summer measurement period. Points are colored based on data density ranging from dark red (high density) to dark blue (low density). N obs is the number of measurements for each pair of sensors.

**Figure 4:**
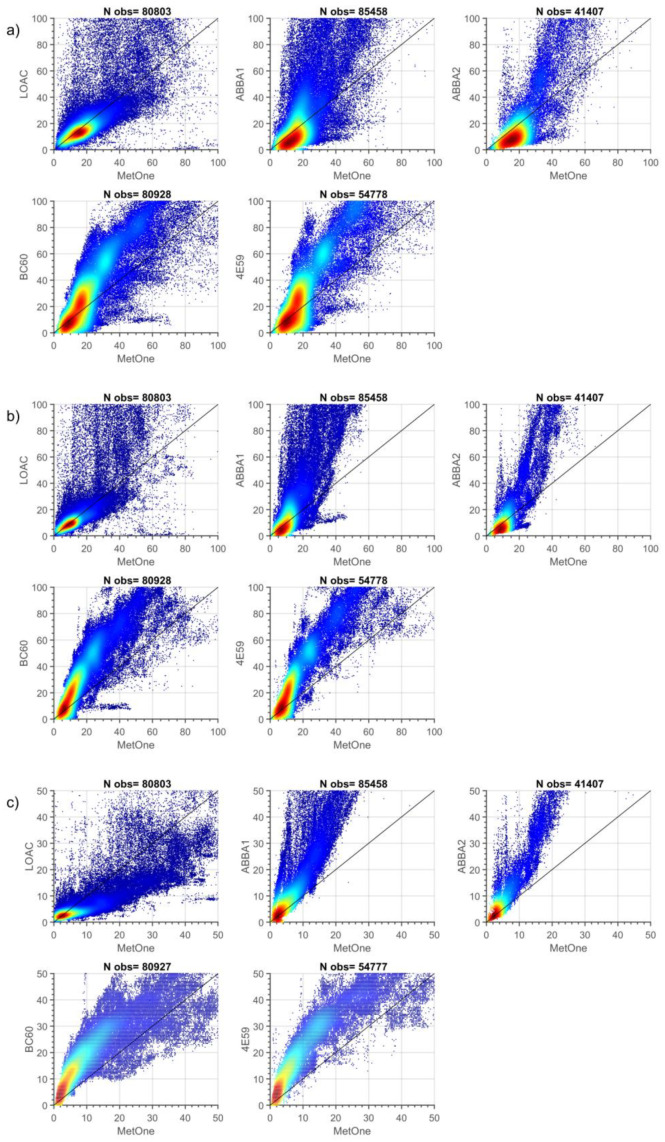
Density scatter plot for of 1-min PM10 (**a**), PM2.5 (**b**) and PM1 (**c**) (µg m^−3^) mass concentrations from the co-located particle sensors during the autumn measurement period. Points are colored based on data density ranging from dark red (high density) to dark blue (low density). N obs is the number of measurements for each pair of sensors.

A better understanding of how data collected from different particle sensors are related derives from the visual inspection of density plots, scatterplots and histograms of 1-min observations during the two measurement campaigns (Figure 3 and Figure 4 and Appendix C).

The comparison of summer data (Figure 3 and Appendix C) highlights even more some patterns previously observed: observations of the two SCKs are very well correlated (R = 0.98, 0.99 and 0.99 respectively for PM10, PM2.5 and PM1) and similarly distributed, while observations from the two OPC-N2s, even though similarly well correlated between each other (R = 0.91, 0.99 and 0.99 respectively for PM10, PM2.5 and PM1) seem to slightly deviate from a linear regression line, indicating some biases between the two sensors though of the same type.

When comparing observations from SCKs and OPC-N2s with those from the MetOne during summer (Figure 3), the density scatterplots show a better agreement for the PM2.5 cut-off. At the same time, lower correlations are observed for PM1 and even worse for PM10. Indeed, low-cost sensors present evident tails in their populations, a feature particularly marked for the two SCKs (4E59 and BC60), in spite of their better correlation with respect to OPC-N2s (ABBA1 and ABBA2) for PM10 and PM2.5. These tails are likely caused by the occurrence of instrumental spikes not removed by the Hampel filter, as observed by data clusters departing from the main data clouds in the scatter plots.

The comparison of autumn data (Figure 4 and Appendix C) shows again that data from the two couples of low-cost sensors are reciprocally well correlated, particularly in the case of the two SCKs (R = 0.91, 0.95 and 0.98 respectively for PM10, PM2.5 and PM1), but also in the case of OPC-N2s for PM2.5. The comparison with observations from the MetOne highlights higher correlations with respect to the summer season for PM1 and PM2.5 for both kinds of low-cost sensors and especially for the OPC-N2s, while for PM10 autumn correlations are higher than the summer ones only for OPC-N2s. Biases and tails are evidenced in the histograms and scatterplots for both types of low-cost sensors, even though clear clusters emerge in the case of the two SCKs. Moreover, all the data are mostly positioned above the bisector, suggesting that owing to the generally higher relative humidity of the autumn season at this latitude and the heating inlet of the MetOne, particle size measured by the sensors is different; the MetOne does not show the effect of the hygroscopic growth, as seen in detail later on. The observations from LOAC, instead, show a different behavior, showing a general tendency to underestimate aerosol mass concentration with respect to the MetOne.

#### 3.1.2. Effect of Time Resolution

**Figure 5:**
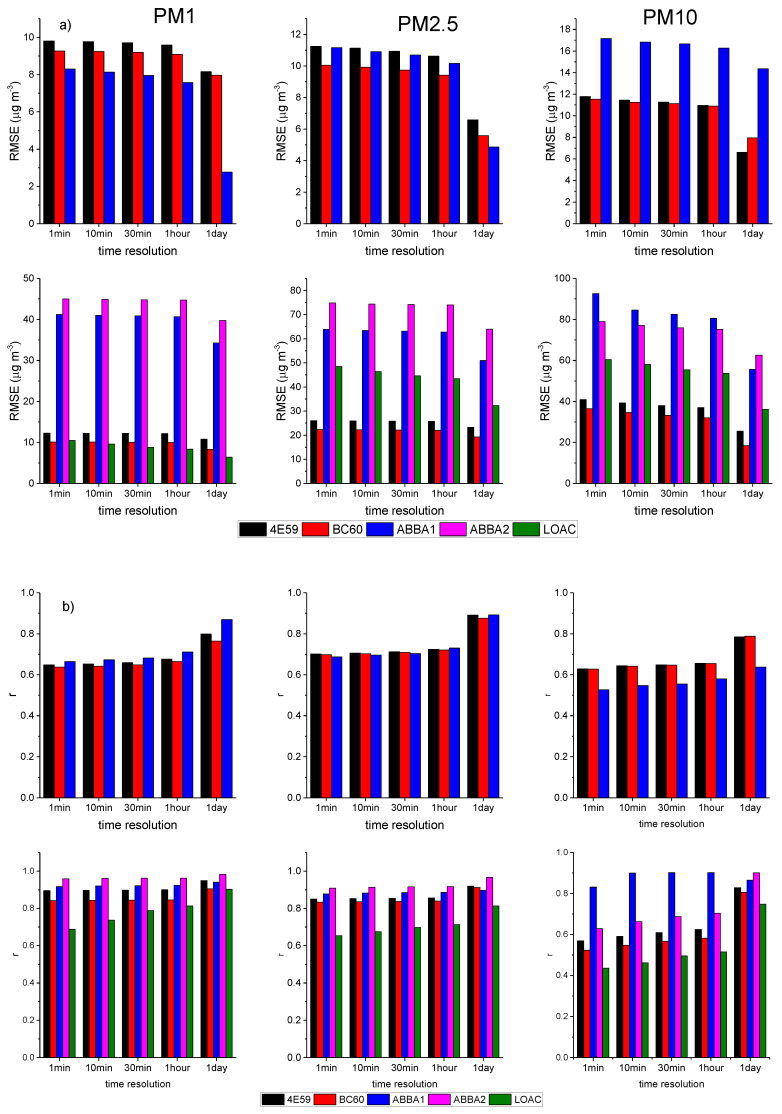
RMSE (root mean square error) (**a**) and r Pearson’s correlation coefficient (**b**) of the optical sensors in measuring PM1, PM2.5 and PM10 mass concentrations under varying time resolution (1 min, 10 min, 30 min, 1 h and 1 day) using the MetOne as the reference sensor and during the two-measurement periods (summer in the upper panel, autumn in the lower panel).

Figure 5 presents the evaluation of the performances of the sensors through the RMSE and the r correlation coefficient at different time resolutions (from 1 min to 1 day) during the two measurement periods, while the complete series of the statistical indicators is presented in Appendix D. During both measurement periods, the values highlight a tendency for the performance of the low-cost sensors to improve with lower time resolutions, as evidenced by the increasingly higher correlation coefficients and the lower biases. The comparison of the summer and autumn values shows that as previously observed, the agreement of the sensors is higher during autumn, though also accompanied by higher biases in this season. The performances of the two couples of low-cost sensors within the brand type are very similar. In general, during both measurement periods, the SCKs are more consistent with MetOne than the OPC-N2s in the case of PM10 concentrations, while OPC-N2s reveal lower biases for PM2.5 and PM1. In addition, the MBE values confirm the SCKs’ tendency to overestimate compared to MetOne; conversely, the OPC-N2s tend to underestimate PM2.5 and PM10 observations during summer. As previously described, the LOAC shows reduced correlations for all size fractions at high time resolution, while correlations greatly improve at lower time resolutions. Finally, the bias for the LOAC is particularly low for PM1, which might indicate its better counting efficiency for the smaller particles.

#### 3.1.3. Effects of Meteorological Conditions

As reported in Section 2, the measurement period included different meteorological conditions typical of the warm and cold seasons in the measurement site. Specifically, with the aim of understanding the effects of different weather conditions on the performances of the optical sensors, we investigated the effects of the prevailing weather conditions based on the meteorological variables observed in situ from the co-located meteorological station and a WMO (World Meteorological Organization) synoptic meteorological station located at the Bologna airport; synoptic weather charts; atmospheric vertical soundings at a nearby meteorological station; drop size distribution observations obtained from a co-located OTT-Parsivel disdrometer, OTT Hydromet GmbH, 87437 Kempten, Germany; and maps of dust transport over the Mediterranean region, as simulated by the BSC-DREAM 8b model from the Barcelona Supercomputing Center (https://ess.bsc.es/bsc-dust-daily-forecast). Briefly, the weather conditions observed were the following:

- Thunderstorm on 9th July 2019;
- Saharan dust transport on 10th July 2019;
- Rain on 7th October 2019;
- Mist on 14th October 2019;
- Fair weather on 20th October 2019;
- Cloudy conditions on 23rd October 2019;
- Fog on 25th October 2019;
- Drizzle on 1st November 2019.

**Figure 6:**
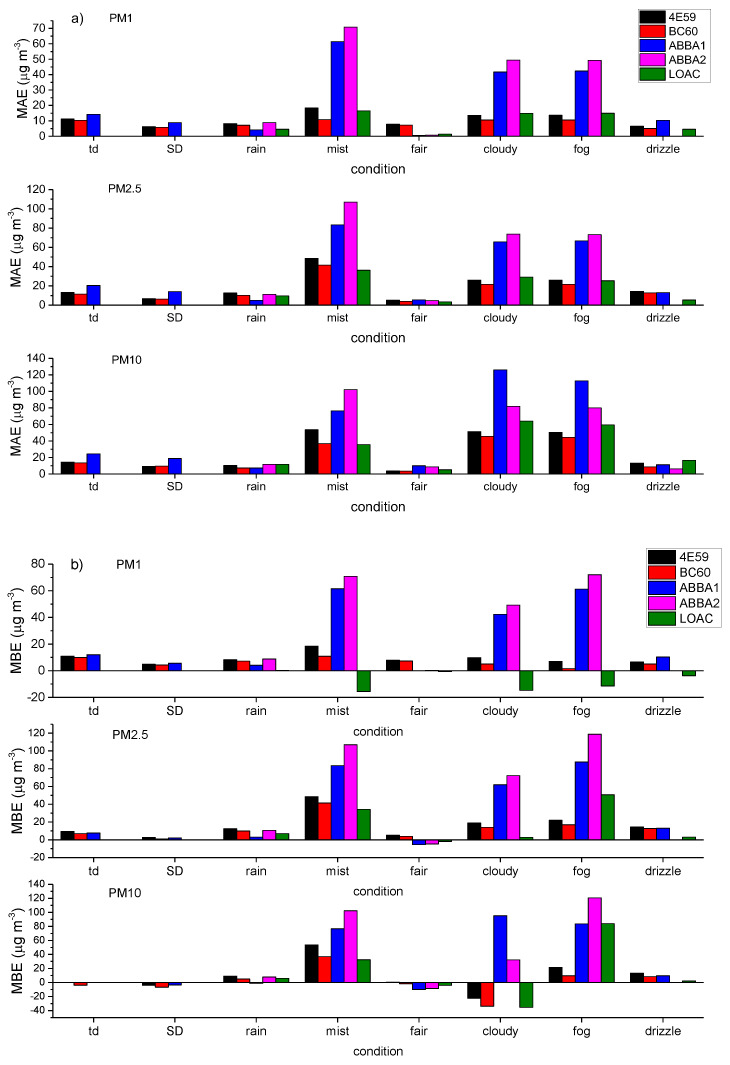

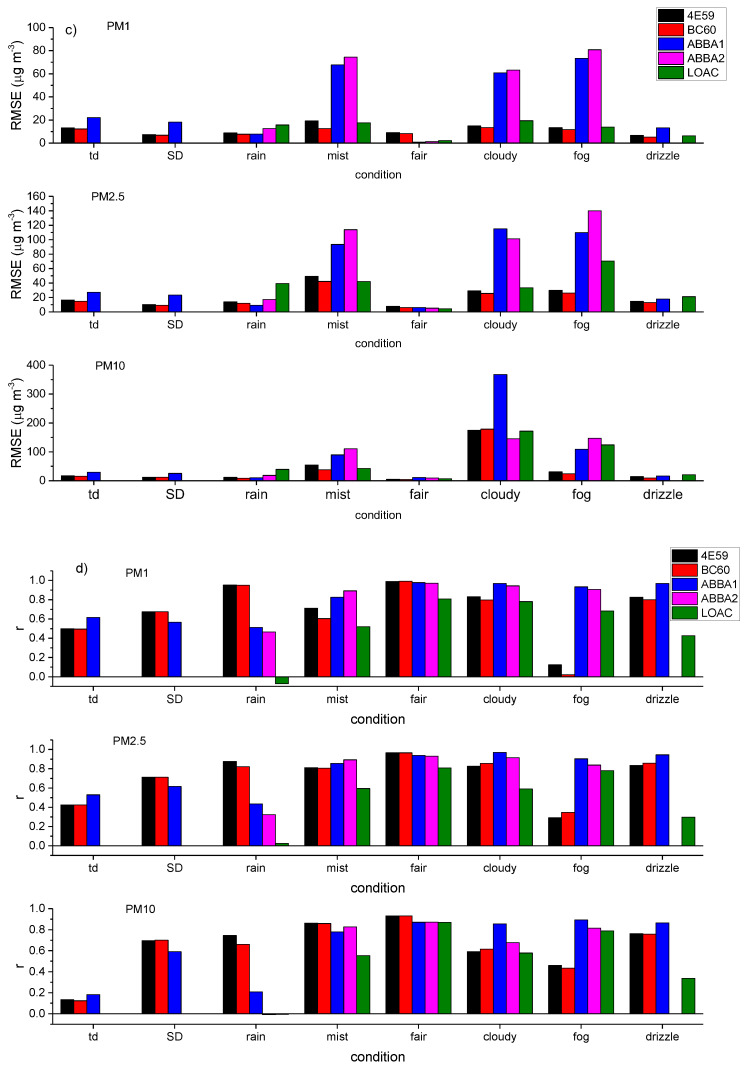
Evaluation of the performances of the different optical sensors in measuring PM1, PM2.5 and PM10 mass concentrations under varying weather conditions (thunderstorm = td, Saharan dust transport = SD, rain, mist, fair weather, cloudy, fog, drizzle) using the MetOne as the reference sensor by calculating the following indexes: (**a**) MAE (mean absolute error); (**b**) MBE (mean bias error); (**c**) RMSE (root mean square error); (**d**) r (Pearson’s correlation coefficient).

Figure 6 presents the performances of the optical sensors during these different weather conditions as evaluated from the calculations of MAE, MBE and RMSE indexes, and correlation coefficients using the MetOne sensor as a reference instrument.

As seen in Figure 6, the performances of all the sensors are the highest during fair weather conditions, when the lowest values of the MAE, MBE and RMSE are reported, associated with high correlation coefficients (>0.80) for all size fractions. Conversely, the performances of the sensors sensibly worsen under mist, cloudy and fog conditions, as expected given the hygroscopic behavior of aerosols. The comparison among the different sensors shows the higher performance of the OPC-N2s and the LOAC in capturing PM1 concentrations under fair weather, while the SCKs perform better for PM2.5 and particularly for PM10 concentrations. Figure 6 shows how the SCKs present a satisfactory, even though never exceptional performance under all weather conditions, while the OPC-N2s and the LOAC reveal very variable behaviors ranging from the excellent performance with fair weather down to fairly biased and poorly correlated under mist, cloudy and fog conditions. The OPC-N2s present high average correlations (>0.6) for most weather conditions except for the Saharan dust transport and rain events. The differentiated behavior of the other sensors may stem from several factors differently affecting particle counts in the various size bins and how they are used in PM assessment. One of the primary factors is basically the different particle size bins and the overall size intervals of each model, leading to an approximation in the instrumental comparison. Secondly, as previously reported, optical sensors are characterized by distinct particle size-selectivity [25]. Thirdly, the use of a constant density factor across the various size ranges to convert number densities to mass concentrations is likely an oversimplification, owing to the intrinsic complexity of PM composition highly inhomogeneous across the size distribution and to the direct and indirect influence of meteorology on particle size and composition [57]. In fact, the enhanced bias among the various sensors observed during the Saharan dust transport should be ascribed to the use of a density factor unsuitable for capturing the prevailing mineral component of the particles associated with this kind of transport. All the other biases instead are attributable mainly to the influence of relative humidity on particle size as a result of variable hygroscopic growth leading to an overestimation of particle mass by the optical sensors with respect to the MetOne sensor endowed with the inlet heater.

**Figure 7:**
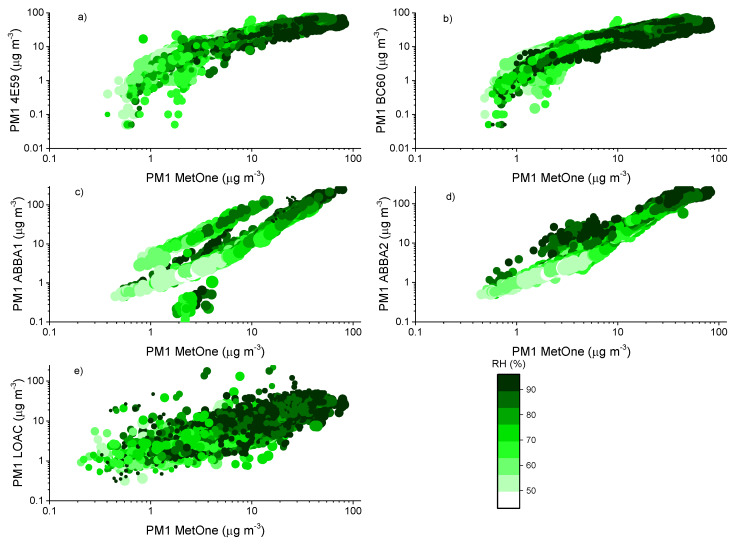
Comparison of 10-min PM1 mass concentrations observed by the MetOne and the other optical sensors: (**a**,**b**) the two SCKs (4E59 and BC60); (**c**,**d**) are the two OPC-N2s (ABBA1 and ABBA2); (**e**) is the LOAC during the autumn measurement period. The color scale indicates the relative humidity (%) value at the time of the measurements, while the size of the marker is proportional to the value of temperature at the time of the measurements.

**Figure 8:**
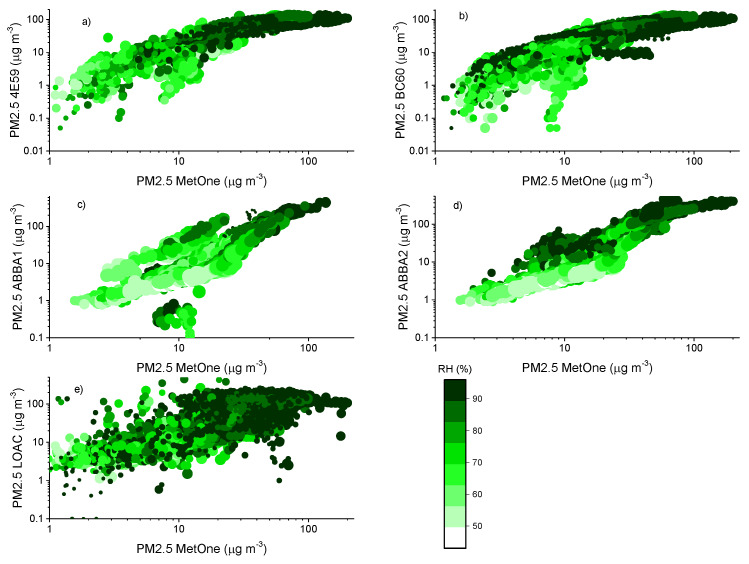
Comparison of 10-min PM2.5 mass concentrations observed by the MetOne and the other optical sensors: (**a**,**b**) are the two SCKs (4E59 and BC60), (**c**,**d**) are the two OPC-N2s (ABBA1 and ABBA2) and (**e**) is the LOAC during the autumn measurement period. The color scale indicates the relative humidity (%) value at the time of the measurements, while the size of the marker is proportional to the value of temperature at the time of the measurements.

**Figure 9:**
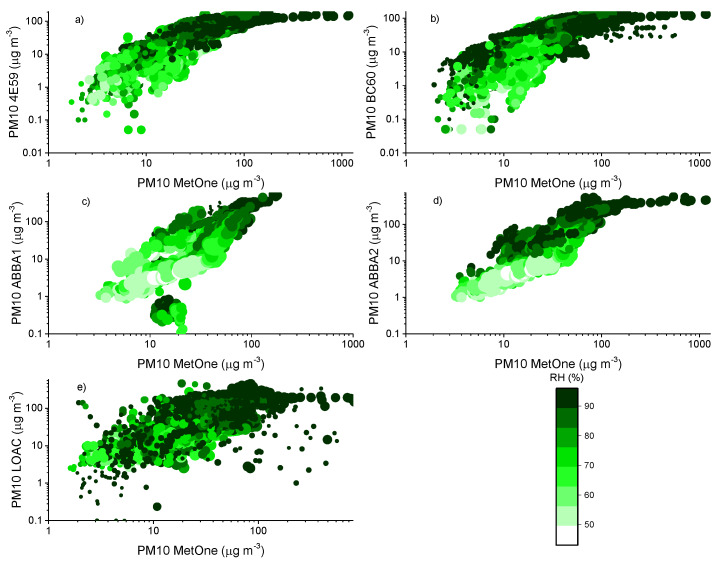
Comparison of 10-min PM10 mass concentrations observed by the MetOne and the other optical sensors: (**a**,**b**) are the two SCKs (4E59 and BC60), (**c**,**d**) are the two OPC-N2s (ABBA1 and ABBA2) and (**e**) is the LOAC during the autumn measurement period. The color scale indicates the relative humidity (%) value at the time of the measurements, while the size of the marker is proportional to the value of temperature at the time of the measurements.

The effect of relative humidity can be better assessed by observing the scatter plots of the aerosol masses obtained for the various devices. Figure 7, Figure 8 and Figure 9 report the comparison of 10-min averaged PM1, PM2.5 and PM10 concentrations of the MetOne vs. those obtained from all the others with different color tones as a function of relative humidity and temperature, during the autumn measurement period (results for the summer measurement period are reported in Appendix E).

During the autumn measurement period, the temperature in Bologna varied between 5 and 28 °C, while relative humidity varied in the range 43–96%. Overall, Figure 5, Figure 6 and Figure 7 highlight the effects of relative humidity conditions on PM concentrations observed by all the optical particle counters. In particular, OPC-N2 sensors tend to overestimate particulate matter concentrations under high relative humidity conditions, while they seem to underestimate PM concentrations under low relative humidity, especially for PM2.5 and PM10. The behavior of the two SCKs seems more coherent and less affected by humidity conditions than that of the OPC-N2s, while the PM10 readings from this sensor type present also the effect of temperature conditions, possibly associated with the protective case used to house the two sensors. The more significant effect of relative humidity conditions on PM2.5 and PM10 concentrations, related with the condensation of water vapor and hygroscopic growth of particles [58], is in agreement with the previous results on the dependence of hygroscopicity on particle size, previously observed by [59]. The hygroscopic growth of particles inevitably affects the response of all the particle counters, resulting in a possible overestimation of the mass because of the reduced molecular mass of water [60]. The differences against the MetOne reference instrument can be ascribed to the thermal dehydration of particles within the instrument body, leading to a decrease in particle size, and therefore, to a shift in the size distribution in this instrument. Since size distribution affects the retrieved mass data through the application of the conversion previously described in the experimental section, the bias between instruments might be larger at higher relative humidity values owing to enhanced hygroscopic growth.

**Figure 10:**
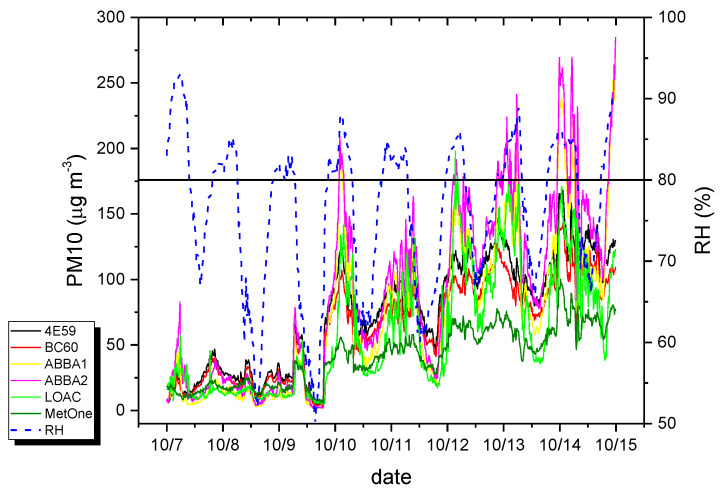
Time series of 10-min PM10 mass concentrations observed by the various optical sensors and relative humidity values during the week of 7–14 October 2019.

Figure 10 presents a focus of the PM10 concentrations and of relative humidity values observed on the week of 7–14 October 2019, enlightening the occurrence of a bias among the sensors. In particular, an overestimation of mass concentrations was observed in the data from low-cost sensors and by the LOAC, when relative humidity was higher than 70–80%, and under fog conditions, in agreement with previous observations [57,61,62], likely due to the use of an inadequate density factor in these conditions.

**Figure 11:**
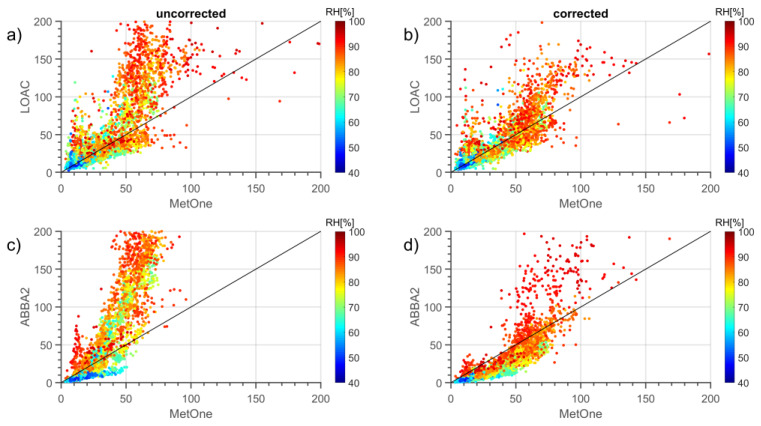
Comparison of 10-min PM10 mass concentrations observed by the MetOne, by one of the two OPC-N2 (ABBA2) and by the LOAC during October 2019, both without (left panels, **a**,**c**) and with (right panels, **b**,**d**) the correction with the hygroscopic growth factor. The color scale indicates the relative humidity (%) value at the time of the measurements.

Under these circumstances of high relative-humidity conditions, we tested the use of the hygroscopic growth factor developed by [45] based on the *ĸ*-Köhler theory. Specifically, the particle diameters of the OPC-N2 and of the LOAC sensors (as reported previously, the SCKs do not provide particle counts explicitly in the various size bins and therefore cannot be subjected to this correction) were corrected by a hygroscopic growth factor [48] to take into account the changes in particle size due to water uptake:

(4) $$g\left(\mathrm{RH}\right)={\left(1+\kappa \frac{\mathrm{RH}}{100-\mathrm{RH}}\right)}^{1/3}$$

(5) $$D_{\mathrm{wet}}=D\cdot g\left(\mathrm{RH}\right)$$

where RH is the relative humidity obtained by the weather station, and *κ* is a parameter that describes the particle hygroscopicity and is assumed to be 0.62, an optimized value for a mixture of organic and inorganic particles in polluted environments fitting Bologna airshed characteristics. “Corrected” particle mass concentrations were then estimated by summing the concentration masses in the various size bins fitting to the respective aerosol cutoff. Figure 11 reports the scatterplots for the PM10 mass concentrations observed by one of the two OPC-N2s (the ABBA2) and the LOAC vs. those observed by the MetOne during October 2019, both with and without the use of the correction with the hygroscopic growth factor.

The comparison of the regression for corrected and uncorrected mass concentrations indicates that the correction performs well and improves the agreement with the MetOne reference instrument, even though in the case of the OPC-N2 sensor seems to lower also the values observed under moderate or low relative humidity conditions. Therefore, it seems useful to adopt a threshold value of RH (Relative Humidity) beyond which the correction can be applied.

### 3.2. Particle Number Densities and Particle Size Distributions

The correlations of particle number densities observed by the instruments were evaluated over three weeks, considering in particular, two periods of weak synoptic forcing respectively in summer (19–24 July 2019) and in winter (5–11 February 2020), characterized by constant high atmospheric pressure, low wind speeds and absence of precipitation, and one in autumn (19–26 October 2019) was characterized by the presence of frequent fog and cloudy conditions. These analyses were carried out by comparing the particle number densities retrieved by the two OPC-N2s and by the LOAC with those collected by the MetOne. In this framework, the SCKs, which might be classified as SSys ready-to-use out of the box systems according to [35], do not provide particle number densities explicitly, and therefore, could not be evaluated against the other ones. Moreover, during the summer week, only one of the OPC-N2 (ABBA1) and the MetOne were operating.

The correlations were evaluated by estimating seven fractions, obtained by summing the proper bins of each instrument: “fr0.5” (considered as the fraction 0.3–0.5 μm), “fr0.7” (0.5–0.7 μm), “fr1” (0.7–1.0 μm), “fr1_2” (1.0–2.0 μm, not present for LOAC), “fr1_3” (1.0–3.0 μm), “fr5” (3.0–5.0 μm) and “fr10” (5.0–10.0 μm). All data were mediated over 10 min, and the Hampel filter presented in the experimental section was applied to remove outliers.

Table 2 summarizes the results in terms of the coefficient of determination (R^2^) and Pearson and Spearman correlation coefficients.

The lower fractions (fr0.5, fr0.7 and fr1) show, in general, higher correlations than the higher ones, as shown by the low R^2^ and correlation coefficients. This is ascribed to the higher mass contribution of the coarse fraction to PM10 in the warm and substantially drier season, but also to the influence of gravity and therefore to the remarkable stochasticity of suspended coarse particles [17,63]. Similar results were reported in other papers focusing on OPC comparison (see for example, [61]).

In the summer week, poor R^2^ and Pearson correlations were observed between MetOne and ABBA1, while the Spearman correlations were higher, in agreement with the non-normal distribution. During the autumn and winter weeks, instead, high (>0.6 and even >0.9 in most cases) Spearman correlation coefficients were observed between all instruments against the MetOne for almost all fractions. The LOAC sensor presents the lowest correlations with the MetOne in terms of R^2^ and Pearson indexes. This is attributed to the frequent spikes present in LOAC data (even if partially removed by the application of the Hampel filter) in all the size classes simultaneously possibly due to electronic noise during the experiment. The Spearman coefficient, indeed, being a non-parametric alternative for evaluating the correlation, shows a better skill in evaluating it in the presence of spikes. ABBA1 and ABBA2 are both strongly correlated to MetOne, particularly considering the Spearman indexes. Moreover, the correlation coefficients between these two OPCs are always higher than 0.9 for all fractions (data not shown), suggesting, as reported before, that instruments of the same manufacturer tend to behave similarly.

**Figure 12:**
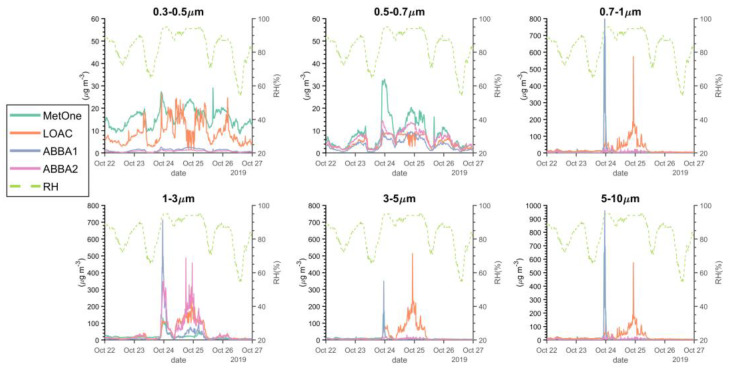
Time series of 1-min particle number densities in the various size fractions (0.3–0.5; 0.5–0.7; 0.7–1; 1–3; 3–5; 5–10 µm) observed by the various optical sensors and relative humidity value during the week of 19–26 October 2019.

Figure 12 shows the time series of particle counts in the different size fractions observed by the two OPC-N2s, the LOAC and the MetOne, together with the corresponding values of relative humidity, during the week of October characterized by frequent fog and cloudy conditions (22–26 October 2019). Particle counts observed by the two OPC-N2s were corrected with the previously cited hygroscopic growth factor. The plots evidence interesting features of the instruments: first of all, the MetOne and the LOAC sensors observe regularly more particles in the smallest size fraction, in agreement with the lower instrumental limit of these two instruments with respect to the OPC-N2 low-cost sensors; secondly, during fog (24th of October) and rain events (24–25 October) and with values of relative humidity close to saturation (95%), the MetOne observes more particles in the second bin than the other sensors; in the coarsest fractions, the LOAC greatly overestimates the number of particles during rain events, but not during fog events, while on the contrary MetOne and ABBA1 seem to perform better under fog than under rainy conditions.

**Figure 13:**
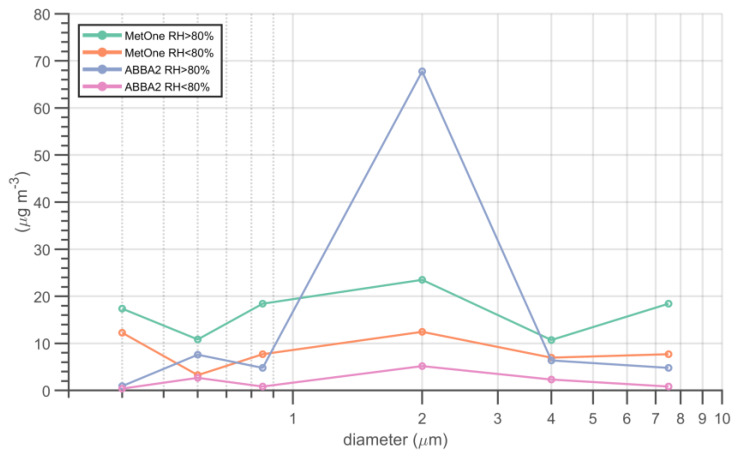
Particle size distributions observed by the MetOne and by one of the two OPC-N2s (ABBA2) for relative humidity values lower and higher than 80% during the week of 19–26 October 2019.

The comparison of particle size distributions observed by the MetOne and one of the two OPC-N2s during the same week of October (Figure 13) shows very clearly that under high relative humidity conditions the disagreement between the two instruments is due to the higher counts of the OPC-N2 in the 1–3 µm size fraction.

## 4. Conclusions

This work describes and discusses the results of an intercomparison exercise that aimed at evaluating the performances of a series of OPCs representative of both research and low-cost devices, the latter becoming extremely popular and extensively used at all scientific levels.

To that end, an intensive long-term experimental field campaign was carried out. The performances of the sensors were evaluated through a robust comprehensive approach considering a mainstream OPC with a heated inlet as the reference instrument, and calculating a series of statistical parameters to capture not only the correlation but also the biases from the reference OPC.

The results of this study indicate that low-cost sensors, and all OPCs, are affected by relevant biases and low correlations when working at elevated time resolution, while the performance improves when lowering the time resolution to hourly or daily averages.

Other biases that emerged from our work are tightly connected with aerosol complexity, and as such, cannot be ignored, since the PM data might be seriously misleading if not considered and suitably corrected. In particular the main deviations were observed using flat density correction factors when converting particle number densities into mass, suggesting a post field campaign reassessment and post-processing of data. This is especially important in countries/areas affected by mineral dust outbreaks whose properties and size distribution spectrum are significantly different from the urban background.

In addition, the performance of a sensor is highly impacted by the prevailing weather conditions, suggesting particular caution in their use for estimating PM concentrations at high relative humidity conditions, such as rain and fog events. Conversely, their performances under conditions of weak synoptic forcing and prevailing anticyclonic conditions were in general characterized by low biases and elevated correlation coefficients. The detailed analysis of the effect of relative humidity suggests/shows that the application of a hygroscopic growth factor to account for the condensation of water vapor on aerosol particles can improve the agreement; however, this approach requires some caution as it may lead to artefacts and shifts in the size distribution which may ultimately result in errors in the estimated PM concentrations.

To conclude, our results show that whatever the application of OPCs is, and the adoption of either research, or in particular, of low-cost PM sensors, the intelligent use of the data is advised based on the following recommendations:

- Data from these devices are precious and extremely informative;
- They can be used reasonably confidently in fair weather conditions and with low time resolution;
- Careful data treatment and evaluation are required in two main cases: airsheds affected by mineral dust, and more generally, during relatively high humidity conditions, rain and fog are observed.

Our results may be extended using longer time series comprehensive of different seasons and analyzing more specifically the effect of PM size and chemical composition on the estimate of PM concentrations from OPCs, taking advantage of the use of ancillary data collected with the suite of co-located instruments. Future research studies focusing on how to automatically adjust data from low-cost sensors under conditions such as high relative humidity or transportation of mineral dust, affecting the observations from optical particle counters may greatly benefit from our results.

## Figures and Tables

**Table 1:** Technical specifications of the MetOne Profiler-212, OPC-N2 from Alphasense, Smart Citizen Kit (SCK), and LOAC (Light Optical Aerosol Counter) optical particle counters.

Instrument	MetOne Profiler-212	OPC-N2	SCK	LOAC
Size (cm)	114.3 × 190.5 + 30.5 for inlet tube	7.5 × 6 × 6.4	6 × 6 × 2	20 × 10 × 5
Weight (g)	1200	<105	65	300
Size range (µm)	0.3–10	0.38–16	0.3–10	0.2–50
Size bins	8 (selectable)	16	3	19
Flow rate (L min^−1^)	1	1.2	≈0.1	≈2
Measurement frequency (s)	1–60	1–5	30	1–60
Laser wavelength (nm)	808	658	680	650
Scattering angle (°)	90	30	90	12 and 60

**Table 2:** Comparison of the particle sensors evaluated by determination coefficients (R^2^), Pearson and Spearman correlation indexes, with respect to the MetOne reference instrument, during the three considered weeks.

**19–24 July 2019**	**ABBA1**								
	**R^2^**	**Pearson**	**Spearman**						
fr0.5	0.481	0.694	0.785						
**fr0.7**	0.480	0.693	0.850						
**fr1**	0.381	0.617	0.688						
**fr1_2**	0.344	0.587	0.617						
**fr1_3**	0.348	0.590	0.632						
**fr5**	0.107	0.328	0.415						
**fr10**	0.0007	0.027	0.041						
**19–26 October 2019**	**ABBA1**			**ABBA2**			**LOAC**		
	**R^2^**	**Pearson**	**Spearman**	**R^2^**	**Pearson**	**Spearman**	**R^2^**	**Pearson**	**Spearman**
**fr0.5**	0.860	0.927	0.971	0.877	0.936	0.966	0.491	0.701	0.866
**fr0.7**	0.867	0.931	0.990	0.812	0.901	0.985	0.723	0.850	0.947
**fr1**	0.769	0.877	0.963	0.651	0.807	0.944	0.557	0.747	0.939
**fr1_2**	0.844	0.919	0.935	0.580	0.762	0.861	--	--	--
**fr1_3**	0.758	0.870	0.893	0.487	0.698	0.803	0.254	0.504	0.803
**fr5**	0.818	0.905	0.833	0.126	0.351	0.776	0.016	0.128	0.724
**fr10**	0.976	0.988	0.651	0.076	0.276	0.571	0.011	0.103	0.713
**5–11 February 2020**	**ABBA1**			**ABBA2**			**LOAC**		
	**R^2^**	**Pearson**	**Spearman**	**R^2^**	**Pearson**	**Spearman**	**R^2^**	**Pearson**	**Spearman**
**fr0.5**	0.819	0.905	0.986	0.752	0.867	0.985	0.460	0.678	0.887
**fr0.7**	0.778	0.882	0.987	0.771	0.878	0.978	0.610	0.781	0.934
**fr1**	0.500	0.707	0.920	0.462	0.680	0.908	0.593	0.770	0.958
**fr1_2**	0.066	0.507	0.966	0.209	0.457	0.953	--	--	--
**fr1_3**	0.252	0.502	0.944	0.203	0.451	0.920	0.130	0.360	0.941
**fr5**	0.803	0.896	0.585	0.841	0.917	0.581	0.328	0.572	0.780
**fr10**	0.931	0.965	0.203	0.843	0.918	0.278	0.533	0.730	0.651

**Figure A1:**
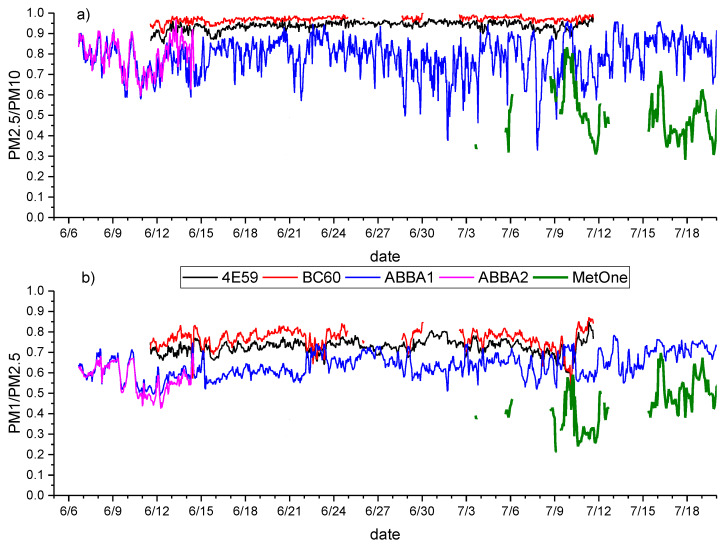
Comparison of hourly PM2.5/PM10 (**a**), and PM1/PM2.5 ratios (**b**) from the co-located particle sensors during the summer measurement period. 4E59 (black line) and BC60 (red line) are the two SCK sensors; ABBA1 (blue line) and ABBA2 (pink line) are the two OPC-N2s sensors. MetOne (thick green line) is the reference instrument.

**Figure A2:**
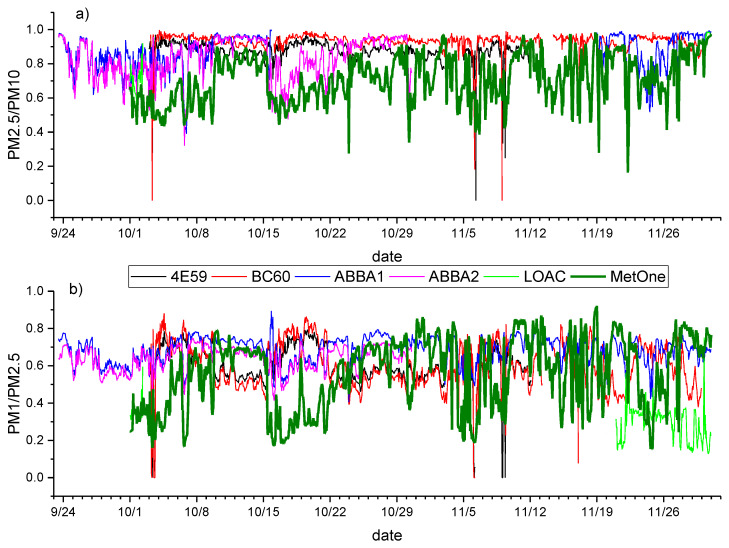
Comparison of hourly PM2.5/PM10 (**a**) and PM1/PM2.5 (**b**) ratios from the co-located particle sensors during the autumn measurement period. 4E59 (black line) and BC60 (red line) are the two SCK sensors; ABBA1 (blue line) and ABBA2 (pink line) are the two OPC-N2s sensors. LOAC is the light green line. MetOne (thick olive green line) is the reference instrument.

**Figure A3:**
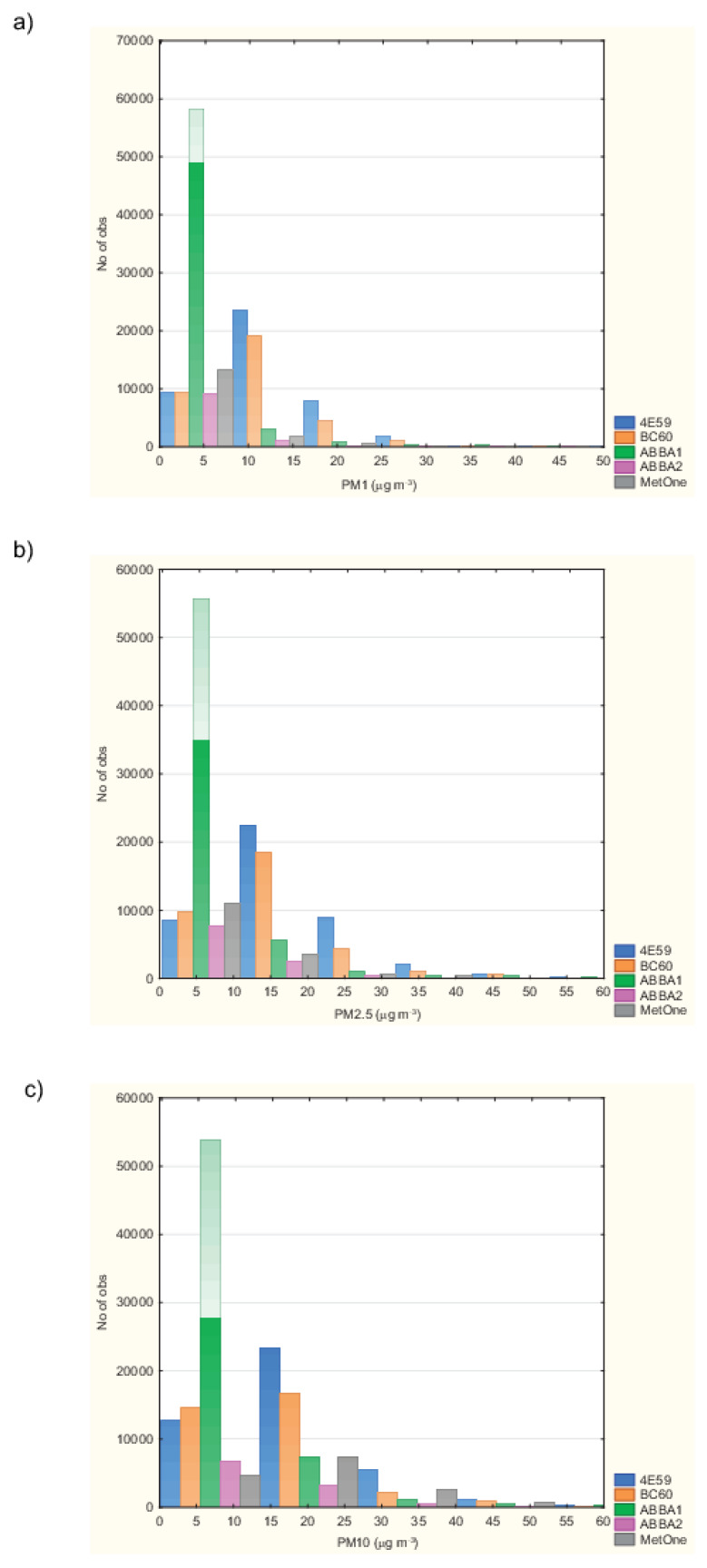
Frequency distributions of 1-min PM1 (**a**), PM2.5 (**b**) and PM10 (**c**) mass concentrations observed by the MetOne (grey bars) and the other optical sensors; i.e., the two SCKs (4E59 and BC60, respectively blue and red bars) and the two OPC-N2 (ABBA1 and ABBA2, respectively green and purple bars) during the summer measurement period.

**Figure A4:**
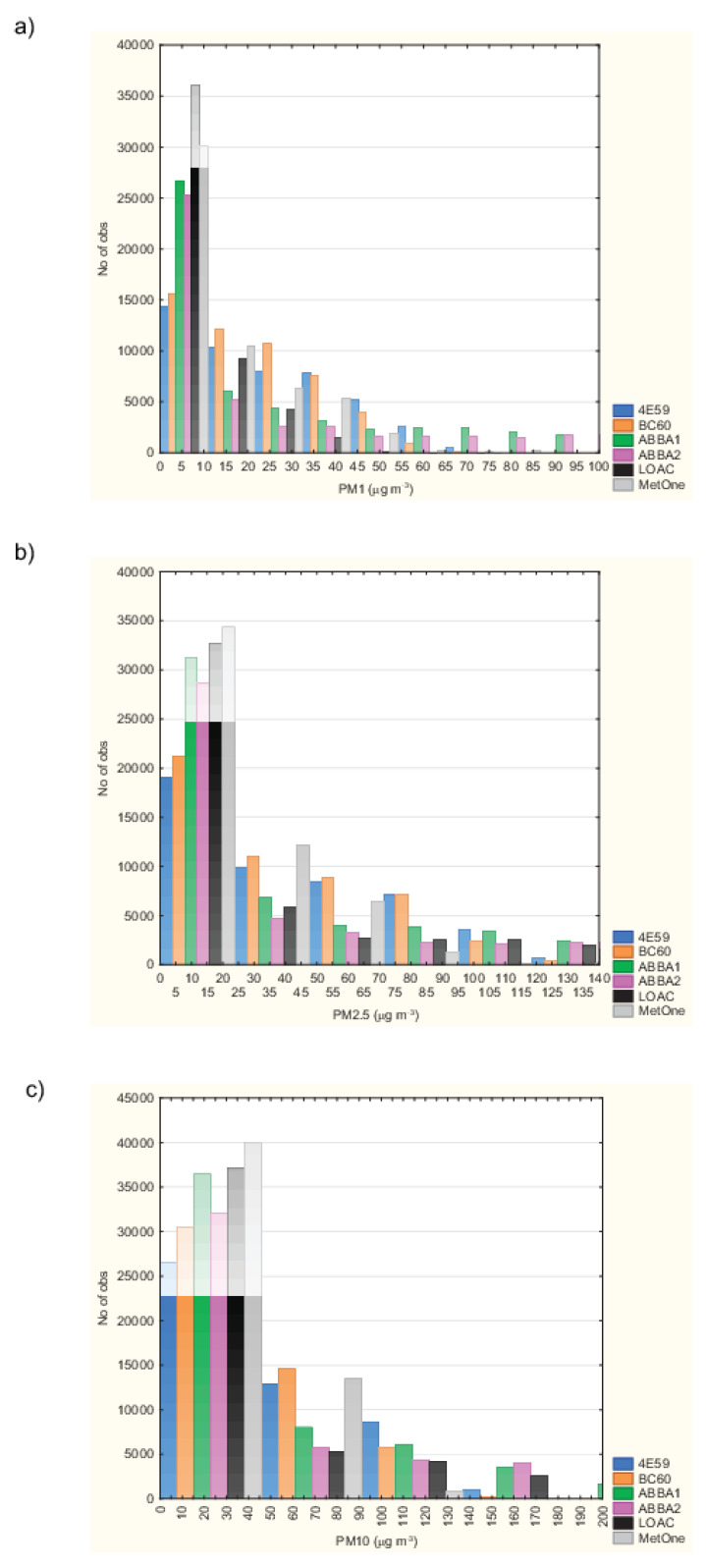
Frequency distributions of 1-min PM1 (**a**), PM2.5 (**b**) and PM10 (**c**) mass concentrations observed by the MetOne (grey bars) and the other optical sensors; i.e., the two SCKs (4E59 and BC60, respectively blue and red bars), the two OPC-N2 (ABBA1 and ABBA2, respectively green and purple bars) and the LOAC (black bars) during the autumn measurement period.

**Figure A5:**
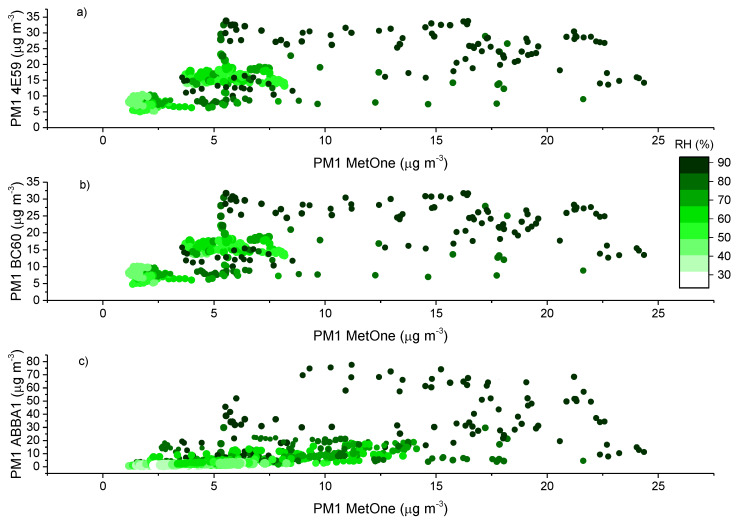
Comparison of 10-min PM1 mass concentrations observed by the MetOne and the other optical sensors: (**a**,**b**) the two SCKs (4E59 and BC60); (**c** one of the two OPC-N2s (ABBA1) during the summer measurement period. The color scale indicates the relative humidity (%) value at the time of the measurements, while the size of the marker is proportional to the value of temperature at the time of the measurements.

**Figure A6:**
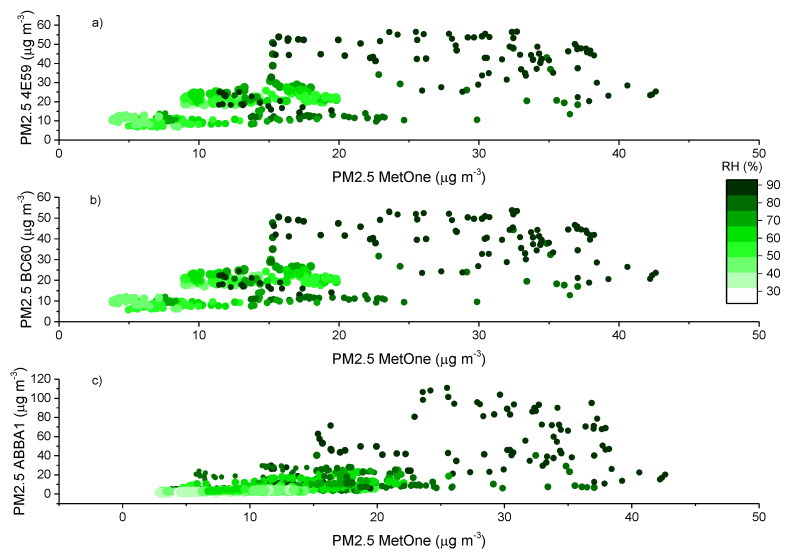
Comparison of 10-min PM2.5 mass concentrations observed by the MetOne and the other optical sensors: (**a**,**b**) the two SCKs (4E59 and BC60); (**c**) one of the two OPC-N2s (ABBA1) during the summer measurement period. The color scale indicates the relative humidity (%) value at the time of the measurements, while the size of the marker is proportional to the value of temperature at the time of the measurements.

**Figure A7:**
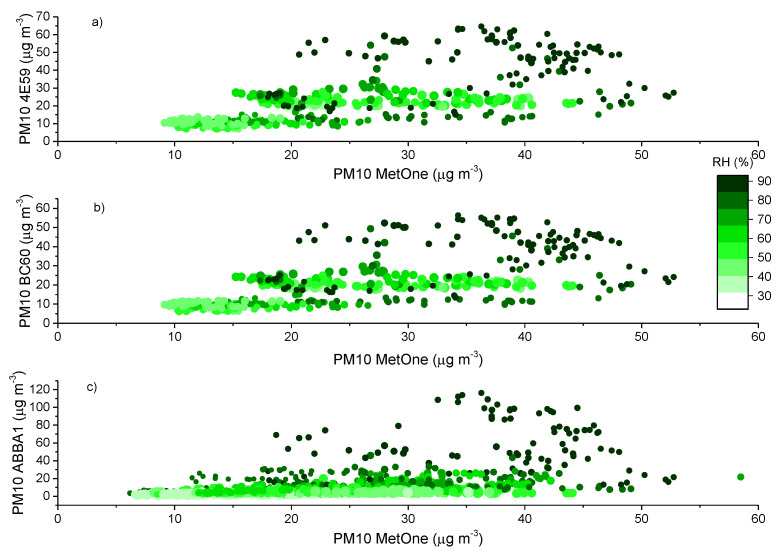
Comparison of 10-min PM10 mass concentrations observed by the MetOne and the other optical sensors: (**a**,**b**) the two SCKs (4E59 and BC60); (**c**) one of the two OPC-N2s (ABBA1 during the summer measurement period. The color scale indicates the relative humidity (%) value at the time of the measurements, while the size of the marker is proportional to the value of temperature at the time of the measurements.

## Appendix A

Here we provide details about and formulas for the statistical parameters used to evaluate the performance of the sensors.

The mean bias error (*MBE*) is used to estimate the average bias in the sensor with respect to the reference instrument and is calculated as:

(A1) $$MBE=\frac{1}{n}\overset{n}{\underset{i=1}{\sum }}\left(P_{i}-O_{i}\right)$$

where *O_i_* is the observation value from the reference sensor (here, MetOne) and *P_i_* is the observation value from the other sensor. A positive bias means that the sensor tends to overestimate the observations from the reference sensor. Conversely, underestimation results in negative biases.

The mean absolute error (*MAE*) measures the average magnitude of the errors in the observations from the sensors with respect to the reference sensor, without considering their direction. It is the average over the test sample of the absolute differences between prediction and actual observation where all individual differences have equal weight, calculated as:

(A2) $$MAE=\frac{1}{n}\overset{n}{\underset{i=1}{\sum }}\left|P_{i}-O_{i}\right|$$

The root mean square error (*RMSE*) is a quadratic scoring rule measuring the average magnitude of the error as the *MBE* and is the square root of the average of squared differences between prediction and actual observation:

(A3) $$RMSE=\sqrt{\frac{1}{n}\overset{n}{\underset{i=1}{\sum }}{\left(P_{i}-O_{i}\right)}^{2}}$$

## Appendix B

Here we present the comparison of hourly observations of PM2.5/PM10 and PM2.5/PM10 ratios recorded by the two SCKs (4E59 and BC60), the two OPC-N2 (ABBA1 and ABBA2), the LOAC and the MetOne reference sensor during the summer and autumn measurement periods.

## Appendix C

Here we present frequency distributions for 1-min particulate matter concentrations observed by the two SCKs (4E59 and BC60), the two OPC-N2 (ABBA1 and ABBA2) and the LOAC during the summer and autumn measurement periods.

## Appendix D

Here we present tables for the performance of the sensors at varying time resolution and during the two measurement periods, as evaluated by the comparison with the observations from the MetOne reference sensor.

**Table A1:** Performances of the particle sensors as evaluated from the comparison with the observations with MetOne reference sensor and from the calculation of a set of statistical parameters (MBE = mean bias error; MAE = mean absolute error; RMSE = root mean square error; NMBE, NMAE and NRMSE are MBE, MAE and RMSE normalized over the observation range, while CVMBE, CVMAE and CVRMSE are normalized over the observation average), at varying time resolution during the summer measurement period.

	**PM1**			**PM2.5**			**PM10**		
**1min**	**4E59**	**BC60**	**ABBA1**	**4E59**	**BC60**	**ABBA1**	**4E59**	**BC60**	**ABBA1**
**mbe**	7.94	7.46	0.49	6.09	4.61	−2.64	−2.33	−4.80	−11.01
**mae**	8.44	8.01	3.42	8.36	7.34	6.70	8.84	8.86	14.26
**rmse**	9.80	9.27	8.30	11.25	10.05	11.16	11.79	11.53	17.17
**nmbe**	0.32	0.30	0.02	0.10	0.08	−0.05	−0.01	−0.02	−0.04
**cvmbe**	1.92	1.80	0.12	0.64	0.49	−0.28	−0.11	−0.23	−0.54
**nmae**	0.34	0.33	0.14	0.14	0.13	0.12	0.03	0.03	0.05
**cvmae**	2.04	1.93	0.83	0.88	0.78	0.71	0.43	0.43	0.70
**nrmse**	0.40	0.38	0.34	0.19	0.17	0.19	0.04	0.04	0.06
**cvrmse**	2.36	2.24	2.00	1.19	1.06	1.18	0.58	0.56	0.84
**pearson**	0.65	0.64	0.66	0.70	0.70	0.69	0.63	0.63	0.53
**Spearman**	0.71	0.70	0.80	0.73	0.73	0.79	0.69	0.69	0.67
	**PM1**			**PM2.5**			**PM10**		
**10min**	**4E59**	**BC60**	**ABBA1**	**4E59**	**BC60**	**ABBA1**	**4E59**	**BC60**	**ABBA1**
**mbe**	7.96	7.49	0.48	6.10	4.63	−2.66	−2.38	−4.83	−11.04
**mae**	8.44	8.02	3.39	8.31	7.29	6.65	8.57	8.62	14.18
**rmse**	9.77	9.24	8.13	11.13	9.93	10.91	11.45	11.25	16.83
**nmbe**	0.34	0.32	0.02	0.11	0.08	−0.05	−0.01	−0.02	−0.04
**cvmbe**	1.92	1.81	0.12	0.64	0.49	−0.28	−0.12	−0.24	−0.54
**nmae**	0.36	0.34	0.14	0.15	0.13	0.12	0.03	0.03	0.05
**cvmae**	2.04	1.93	0.82	0.88	0.77	0.70	0.42	0.42	0.69
**nrmse**	0.41	0.39	0.34	0.20	0.18	0.20	0.04	0.04	0.06
**cvrmse**	2.36	2.23	1.96	1.18	1.05	1.15	0.56	0.55	0.82
**pearson**	0.65	0.64	0.67	0.71	0.70	0.70	0.64	0.64	0.55
**Spearman**	0.71	0.70	0.81	0.74	0.73	0.81	0.71	0.70	0.71
	**PM1**			**PM2.5**			**PM10**		
**30min**	**4E59**	**BC60**	**ABBA1**	**4E59**	**BC60**	**ABBA1**	**4E59**	**BC60**	**ABBA1**
**mbe**	7.97	7.51	0.45	6.09	4.63	−2.72	−2.55	−4.97	−11.14
**mae**	8.43	8.01	3.34	8.25	7.23	6.60	8.44	8.53	14.19
**rmse**	9.71	9.20	7.96	10.94	9.74	10.70	11.26	11.12	16.67
**nmbe**	0.34	0.32	0.02	0.11	0.09	−0.05	−0.01	−0.02	−0.04
**cvmbe**	1.92	1.81	0.11	0.64	0.49	−0.29	−0.12	−0.24	−0.54
**nmae**	0.36	0.34	0.14	0.15	0.13	0.12	0.03	0.03	0.06
**cvmae**	2.03	1.93	0.81	0.87	0.76	0.70	0.41	0.42	0.69
**nrmse**	0.41	0.39	0.34	0.20	0.18	0.20	0.04	0.04	0.07
**cvrmse**	2.34	2.22	1.92	1.15	1.03	1.13	0.55	0.54	0.81
**pearson**	0.66	0.65	0.68	0.71	0.71	0.70	0.65	0.65	0.55
**Spearman**	0.71	0.70	0.81	0.74	0.73	0.81	0.71	0.71	0.72
	**PM1**			**PM2.5**			**PM10**		
**1hour**	**4E59**	**BC60**	**ABBA1**	**4E59**	**BC60**	**ABBA1**	**4E59**	**BC60**	**ABBA1**
**mbe**	7.97	7.53	0.42	6.05	4.60	−2.77	−2.68	−5.07	−11.22
**mae**	8.34	7.94	3.28	8.16	7.12	6.50	8.25	8.25	14.02
**rmse**	9.58	9.10	7.58	10.62	9.42	10.16	10.96	10.89	16.27
**nmbe**	0.35	0.33	0.02	0.13	0.10	−0.06	−0.01	−0.02	−0.05
**cvmbe**	1.92	1.82	0.10	0.64	0.49	−0.29	−0.13	−0.25	−0.55
**nmae**	0.37	0.35	0.14	0.18	0.16	0.14	0.04	0.04	0.07
**cvmae**	2.01	1.91	0.79	0.86	0.75	0.68	0.40	0.40	0.68
**nrmse**	0.42	0.40	0.33	0.24	0.21	0.22	0.05	0.05	0.08
**cvrmse**	2.31	2.19	1.83	1.12	0.99	1.07	0.53	0.53	0.79
**pearson**	0.68	0.66	0.71	0.72	0.72	0.73	0.66	0.65	0.58
**Spearman**	0.71	0.69	0.82	0.73	0.73	0.81	0.70	0.69	0.73
	**PM1**			**PM2.5**			**PM10**		
**1day**	**4E59**	**BC60**	**ABBA1**	**4E59**	**BC60**	**ABBA1**	**4E59**	**BC60**	**ABBA1**
**mbe**	7.77	7.57	−0.22	5.91	4.78	−3.87	−4.62	−6.49	−12.99
**mae**	7.77	7.57	1.96	5.91	4.78	4.36	5.08	6.58	12.99
**rmse**	8.16	7.97	2.74	6.58	5.58	4.86	6.62	7.96	14.35
**nmbe**	0.91	0.89	−0.03	0.30	0.25	−0.20	−0.18	−0.25	−0.50
**cvmbe**	1.88	1.83	−0.05	0.62	0.50	−0.40	−0.22	−0.31	−0.62
**nmae**	0.91	0.89	0.23	0.30	0.25	0.22	0.20	0.26	0.50
**cvmae**	1.88	1.83	0.48	0.62	0.50	0.46	0.24	0.32	0.62
**nrmse**	0.96	0.93	0.32	0.34	0.29	0.25	0.26	0.31	0.56
**cvrmse**	1.98	1.93	0.67	0.69	0.58	0.51	0.32	0.38	0.69
**pearson**	0.80	0.76	0.87	0.89	0.88	0.89	0.79	0.79	0.64
**Spearman**	0.80	0.80	0.90	0.83	0.78	0.83	0.73	0.70	0.60

**Table A2:** Performances of the particle sensors as evaluated from the comparison with the observations with the MetOne reference sensor and from the calculation of a set of statistical parameters (MBE = mean bias error; MAE = mean absolute error; RMSE = root mean square error; NMBE, NMAE and NRMSE are MBE, MAE and RMSE normalized over the observation range, while CVMBE, CVMAE and CVRMSE are normalized over the observation average), at varying time resolution during the autumn measurement period.

	**PM1**					**PM2.5**					**PM10**				
**1min**	**4E59**	**BC60**	**ABBA1**	**ABBA2**	**LOAC**	**4E59**	**BC60**	**ABBA1**	**ABBA2**	**LOAC**	**4E59**	**BC60**	**ABBA1**	**ABBA2**	**LOAC**
**mbe**	9.23	6.53	24.63	26.97	−3.73	17.84	14.93	34.14	39.73	19.55	15.30	9.08	34.42	34.99	19.23
**mae**	9.95	8.07	24.83	27.13	6.05	19.77	17.05	37.04	43.15	23.98	22.38	18.13	41.29	44.49	28.28
**rmse**	12.30	10.12	41.21	45.02	10.47	26.01	22.30	63.88	74.81	48.45	40.87	36.49	92.58	79.01	60.42
**nmbe**	0.11	0.08	0.29	0.32	−0.04	0.08	0.07	0.16	0.18	0.09	0.01	0.01	0.02	0.02	0.01
**cvmbe**	0.67	0.47	1.78	1.95	−0.27	0.79	0.66	1.52	1.77	0.87	0.49	0.29	1.10	1.12	0.61
**nmae**	0.12	0.10	0.30	0.32	0.07	0.09	0.08	0.17	0.20	0.11	0.01	0.01	0.02	0.03	0.02
**cvmae**	0.72	0.58	1.79	1.96	0.44	0.88	0.76	1.65	1.92	1.07	0.71	0.58	1.32	1.42	0.90
**nrmse**	0.15	0.12	0.49	0.54	0.12	0.12	0.10	0.29	0.34	0.22	0.02	0.02	0.06	0.05	0.04
**cvrmse**	0.89	0.73	2.97	3.25	0.76	1.16	0.99	2.84	3.33	2.15	1.30	1.16	2.95	2.52	1.93
**pearson**	0.89	0.84	0.92	0.96	0.69	0.85	0.83	0.88	0.91	0.65	0.57	0.52	0.83	0.63	0.44
**Spearman**	0.96	0.92	0.94	0.98	0.86	0.93	0.91	0.89	0.95	0.84	0.87	0.84	0.83	0.92	0.81
	**PM1**					**PM2.5**					**PM10**				
**10min**	**4E59**	**BC60**	**ABBA1**	**ABBA2**	**LOAC**	**4E59**	**BC60**	**ABBA1**	**ABBA2**	**LOAC**	**4E59**	**BC60**	**ABBA1**	**ABBA2**	**LOAC**
**mbe**	9.23	6.53	24.63	26.96	−3.74	17.83	14.93	34.12	39.72	19.49	15.28	9.08	34.37	34.99	19.14
**mae**	9.93	8.05	24.80	27.10	5.91	19.72	17.01	37.00	43.11	23.57	22.24	18.01	41.02	44.07	27.70
**rmse**	12.24	10.08	40.99	44.88	9.62	25.91	22.21	63.41	74.39	46.32	39.28	34.64	84.55	77.07	58.00
**nmbe**	0.11	0.08	0.30	0.33	−0.05	0.09	0.07	0.17	0.20	0.10	0.01	0.01	0.03	0.03	0.02
**cvmbe**	0.67	0.47	1.78	1.95	−0.27	0.79	0.66	1.52	1.77	0.87	0.49	0.29	1.10	1.12	0.61
**nmae**	0.12	0.10	0.30	0.33	0.07	0.10	0.09	0.19	0.22	0.12	0.02	0.02	0.03	0.04	0.02
**cvmae**	0.72	0.58	1.79	1.96	0.43	0.88	0.76	1.65	1.92	1.05	0.71	0.57	1.31	1.41	0.88
**nrmse**	0.15	0.12	0.50	0.54	0.12	0.13	0.11	0.32	0.37	0.23	0.03	0.03	0.07	0.07	0.05
**cvrmse**	0.88	0.73	2.96	3.24	0.69	1.15	0.99	2.82	3.31	2.06	1.25	1.11	2.70	2.46	1.85
**pearson**	0.90	0.84	0.92	0.96	0.74	0.85	0.84	0.88	0.91	0.68	0.59	0.55	0.90	0.66	0.46
**Spearman**	0.96	0.92	0.94	0.98	0.87	0.93	0.91	0.89	0.95	0.85	0.88	0.85	0.83	0.93	0.82
	**PM1**					**PM2.5**					**PM10**				
**30min**	**4E59**	**BC60**	**ABBA1**	**ABBA2**	**LOAC**	**4E59**	**BC60**	**ABBA1**	**ABBA2**	**LOAC**	**4E59**	**BC60**	**ABBA1**	**ABBA2**	**LOAC**
**mbe**	9.22	6.52	24.63	26.96	−3.74	17.81	14.92	34.13	39.72	19.50	15.27	9.08	34.43	34.99	19.16
**mae**	9.91	8.03	24.79	27.09	5.80	19.68	16.97	36.97	43.07	23.25	22.15	17.94	41.01	43.81	27.16
**rmse**	12.20	10.04	40.86	44.80	8.79	25.83	22.14	63.15	74.21	44.59	37.99	33.17	82.54	75.86	55.40
**nmbe**	0.11	0.08	0.30	0.33	−0.05	0.09	0.08	0.18	0.21	0.10	0.02	0.01	0.04	0.04	0.02
**cvmbe**	0.67	0.47	1.78	1.95	−0.27	0.79	0.66	1.52	1.77	0.87	0.49	0.29	1.10	1.12	0.61
**nmae**	0.12	0.10	0.30	0.33	0.07	0.10	0.09	0.20	0.23	0.12	0.02	0.02	0.05	0.05	0.03
**cvmae**	0.72	0.58	1.79	1.96	0.42	0.87	0.75	1.64	1.92	1.03	0.71	0.57	1.31	1.40	0.87
**nrmse**	0.15	0.12	0.50	0.55	0.11	0.14	0.12	0.34	0.40	0.24	0.04	0.04	0.09	0.08	0.06
**cvrmse**	0.88	0.72	2.95	3.23	0.63	1.15	0.98	2.81	3.30	1.98	1.21	1.06	2.63	2.42	1.77
**pearson**	0.90	0.84	0.92	0.96	0.79	0.85	0.84	0.88	0.92	0.70	0.61	0.57	0.90	0.69	0.50
**Spearman**	0.96	0.92	0.94	0.98	0.87	0.93	0.91	0.89	0.95	0.85	0.88	0.85	0.83	0.93	0.83
	**PM1**					**PM2.5**					**PM10**				
**1hour**	**4E59**	**BC60**	**ABBA1**	**ABBA2**	**LOAC**	**4E59**	**BC60**	**ABBA1**	**ABBA2**	**LOAC**	**4E59**	**BC60**	**ABBA1**	**ABBA2**	**LOAC**
**mbe**	9.23	6.53	24.63	26.96	−3.74	17.82	14.91	34.12	39.73	19.49	15.28	9.06	34.41	35.00	19.15
**mae**	9.90	8.02	24.78	27.08	5.66	19.61	16.90	36.92	43.03	22.96	22.07	17.76	40.90	43.62	26.80
**rmse**	12.15	9.99	40.67	44.69	8.39	25.74	22.04	62.76	74.02	43.45	36.94	31.99	80.60	75.15	53.73
**nmbe**	0.12	0.08	0.31	0.34	−0.05	0.10	0.08	0.19	0.22	0.11	0.02	0.01	0.04	0.04	0.02
**cvmbe**	0.67	0.47	1.78	1.95	−0.27	0.79	0.66	1.52	1.77	0.87	0.49	0.29	1.10	1.12	0.61
**nmae**	0.12	0.10	0.31	0.34	0.07	0.11	0.09	0.20	0.24	0.13	0.03	0.02	0.05	0.05	0.03
**cvmae**	0.71	0.58	1.79	1.95	0.41	0.87	0.75	1.64	1.91	1.02	0.70	0.57	1.31	1.39	0.86
**nrmse**	0.15	0.12	0.51	0.56	0.10	0.14	0.12	0.35	0.41	0.24	0.05	0.04	0.10	0.09	0.07
**cvrmse**	0.88	0.72	2.94	3.23	0.61	1.14	0.98	2.79	3.29	1.93	1.18	1.02	2.57	2.40	1.71
**pearson**	0.90	0.85	0.92	0.96	0.81	0.86	0.84	0.89	0.92	0.71	0.62	0.58	0.90	0.70	0.52
**Spearman**	0.96	0.92	0.94	0.98	0.87	0.93	0.91	0.89	0.95	0.86	0.88	0.85	0.84	0.93	0.83
	**PM1**					**PM2.5**					**PM10**				
**1day**	**4E59**	**BC60**	**ABBA1**	**ABBA2**	**LOAC**	**4E59**	**BC60**	**ABBA1**	**ABBA2**	**LOAC**	**4E59**	**BC60**	**ABBA1**	**ABBA2**	**LOAC**
**mbe**	9.30	6.67	24.62	26.99	−3.73	18.04	15.05	34.11	39.75	19.50	15.73	9.27	34.38	35.00	19.16
**mae**	9.32	6.99	24.64	26.99	4.32	18.48	15.64	35.45	41.63	20.75	18.54	14.25	38.05	40.16	23.10
**rmse**	10.80	8.33	34.28	39.74	6.41	23.26	19.34	50.92	64.00	32.33	25.50	18.47	55.65	62.53	36.14
**nmbe**	0.23	0.17	0.62	0.68	−0.09	0.28	0.23	0.53	0.61	0.30	0.17	0.10	0.36	0.37	0.20
**cvmbe**	0.67	0.48	1.78	1.95	−0.27	0.80	0.67	1.52	1.77	0.87	0.50	0.30	1.10	1.12	0.61
**nmae**	0.23	0.17	0.62	0.68	0.11	0.29	0.24	0.55	0.64	0.32	0.20	0.15	0.40	0.42	0.24
**cvmae**	0.67	0.50	1.78	1.95	0.31	0.82	0.70	1.58	1.85	0.92	0.59	0.45	1.21	1.28	0.74
**nrmse**	0.27	0.21	0.86	0.99	0.16	0.36	0.30	0.79	0.99	0.50	0.27	0.19	0.59	0.66	0.38
**cvrmse**	0.78	0.60	2.47	2.87	0.46	1.03	0.86	2.26	2.85	1.44	0.81	0.59	1.78	2.00	1.15
**pearson**	0.95	0.91	0.94	0.98	0.90	0.92	0.91	0.90	0.97	0.81	0.83	0.81	0.87	0.90	0.75
**Spearman**	0.96	0.92	0.93	0.99	0.92	0.94	0.93	0.88	0.95	0.85	0.88	0.87	0.82	0.92	0.80

## Appendix E

Here we present the comparison of 10-min particulate matter concentrations observed from the MetOne and from the two SCKs (4E59 and BC60) and one of the two OPC-N2 (ABBA1) as a function of temperature and relative humidity conditions during the summer measurement period.
